# Similarities of Variant Creutzfeldt-Jakob Disease Strain in Mother and Son in Spain to UK Reference Case

**DOI:** 10.3201/eid2309.170159

**Published:** 2017-09

**Authors:** Abigail B. Diack, Aileen Boyle, Diane Ritchie, Chris Plinston, Dorothy Kisielewski, Jesús de Pedro-Cuesta, Alberto Rábano, Robert G. Will, Jean C. Manson

**Affiliations:** The Roslin Institute, Easter Bush, Scotland, UK (A.B. Diack, A. Boyle, C. Plinston, D. Kisielewski, J.C. Manson);; University of Edinburgh, Edinburgh, Scotland, UK (D. Ritchie, R.G. Will);; Carlos III Institute of Health, Madrid, Spain (J. de Pedro-Cuesta, A. Rábano)

**Keywords:** prions, strain, variant Creutzfeldt-Jakob disease, vCJD, BSE, transmissible spongiform encephalopathy, Spain, United Kingdom, TSE, mouse study

## Abstract

We investigated transmission characteristics of variant Creutzfeldt-Jakob disease in a mother and son from Spain. Despite differences in patient age and disease manifestations, we found the same strain properties in these patients as in UK vCJD cases. A single strain of agent appears to be responsible for all vCJD cases to date.

In 2008 in Spain, 2 cases of variant Creutzfeldt-Jakob disease (vCJD) in first-degree relatives were identified. After the death of a 41-year-old man (patient 1) from vCJD, his 64-year-old mother (patient 2) began showing symptoms of anxiety and depression and, 2 months later, a gait disorder and progressive dementia. Although the clinical duration was relatively short and the early symptoms uncommon in comparison to vCJD cases in the United Kingdom, the overall clinical phenotype and posterior thalamic hyperintensities as seen in an MRI brain scan led to a diagnosis of suspected vCJD. Neuropathological examination confirmed the diagnosis of vCJD. Both patients were 129MM homozygous, had never received a blood transfusion or tissue graft, and had lived in the same town within the Castilla-León region of Spain (Table 1) (*1*). The region is a farming area at high risk for bovine spongiform encephalopathy (BSE); 3 of the 5 cases of vCJD reported in Spain came from this region (*1*). The patients had similar eating habits, which included ingestion of bovine brain. We conducted a study to determine whether these 2 vCJD cases were caused by the BSE agent, whether the agent strain was similar to previously characterized human vCJD cases, and whether the age of the patients would influence the strain characteristics.

**Table 1:** Demographic and clinical features of 2 case-patients from Spain with variant CJD and reference cases from the United Kingdom*

Characteristic	Patient 1	Patient 2	UK cases, n = 150
Patient sex	M	F	
Case-patient age at illness onset, y	41	64	29 (mean)
Case-patient age at death, y	41	64	30 (mean)
Disease duration, mo	9	7	14 (mean)
Early visual symptoms	+	–	6%
Early unsteadiness	–	+	11%
No typical appearance of sporadic CJD on EEG^†^	+	+	100%
Bilateral symmetric pulvinar high signal on MRI scan of brain	Yes	Yes	93%
Positive tonsil biopsy result	ND	ND	19%
History of travel to or residence in United Kingdom	No	No	100%
Codon 129MM	Yes	Yes	100%†
Type 2B PrP	Yes	Yes	100%†

*CJD, Creutzfeldt-Jakob disease; EEG, electroencephalogram; MRI, magnetic resonance imaging; PrP, prion protein; ND, not done; –, negative; +, positive. †Of those tested.

## The Study

We challenged cohorts of mice (RIII, C57BL/6J, and VM) with frozen central nervous system tissue from the 2 patients from Spain and 1 patient originating from the United Kingdom (Table 1) (*2*). The Lothian NHS Board Research Ethics Committee provided ethical consent for the use of the UK material for research; the vCJD tissue samples from Spain were provided by NEIKER-Tecnalia (Derio, Spain). We conducted inoculation, clinical scoring, and neuropathological and biochemical analysis of the mice as previously described (*3**–**5*). Animal studies were conducted according to the regulations of the UK Home Office Animals (Scientific Procedures) Act 1986.

**Figure 1:**
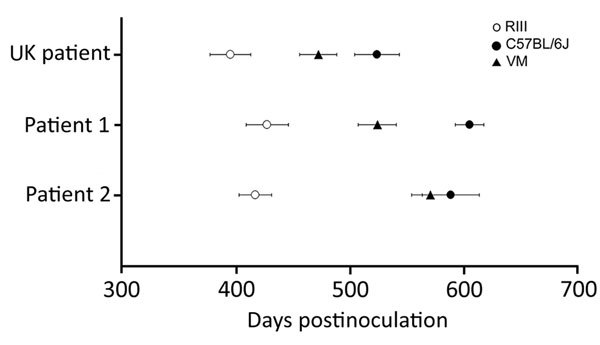
Comparison of vCJD incubation periods in wild-type mice from inoculation of brain tissue homogenates from 2 patients from Spain (son, patient 1; mother, patient 2) with vCJD and a reference patient from the United Kingdom. Results show similar incubation period ranking. Incubation periods were calculated in mice showing clinical and pathologic signs of transmissible spongiform encephalopathy disease. There was a single positive case in VM mice from patient 2. Data show mean incubation period ± SEM. vCJD, variant Creutzfeldt-Jakob disease.

All 3 vCJD brain isolates transmitted successfully, with the appearance of clinical and pathological signs associated with spongiform encephalopathy transmission (Table 2). Inocula from patients 1 and 2 showed the same temporal order of clinical endpoint in each mouse line when compared with inocula from the UK case (Figure 1). We observed a wide range of incubation periods for each mouse line both within and between inocula (Table 2), which is not unusual in primary transmissions.

**Table 2:** Results of inoculation of brain tissue homogenates from 2 patients from Spain with vCJD and a reference patient from the United Kingdom into a panel of wild-type mice*

Brain inoculum source and mouse line	No. mice positive/no. total		Incubation period, d, ± SEM (range)
	Clinical signs of prion disease	Vacuolar pathology	
UK reference case			
RIII	10/15	10/15	395.3 ± 17.9 (295–489)
C57BL/6	13/17	15/17	523.7 ± 19.7 (372–637)
VM	13/16	14/16	472.2 ± 16.1 387–552
Patient 1			
RIII	14/17	15/17	417 ± 14.2 (336–516)
C57BL/6	12/18	12/18	588.4 ± 25.1 (405–706)
VM	7/18	11/18	472.2 ± 16.1 (387–552)
Patient 2			
RIII	12/16	13/16	427.5 ± 18.4 (323–547)
C57BL/6	9/18	11/18	604.9 ± 12.4 (567–692)
VM	4/16	7/16	524 ± 16.8 (501–573)

*Bold indicates significant difference (p<0.05) when compared to the mouse line challenged with UK vCJD. vCJD, variant Creutzfeldt-Jakob disease.

**Figure 2:**
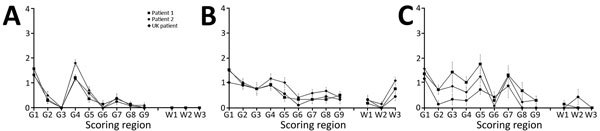
Vacuolation profile of vCJD in wild-type mice from inoculation of brain tissue homogenates from 2 patients from Spain (son, patient 1; mother, patient 2) with vCJD and a reference patient from the United Kingdom. A) RIII mice; B) C57BL/6J mice; C) VM mice. Profiles show similarities in vacuolar pathology intensity and distribution in wild-type mouse brains. Data show mean ± SEM of clinical and pathologic positive mice, with the exception of VM mice arising from the Spain patients, which also include pathologic positive only mice (n≥6 per group). G1–G9, gray matter scoring regions: G1, medulla; G2, cerebellum; G3, superior colliculus; G4, hypothalamus; G5, thalamus; G6, hippocampus; G7, septum; G8, retrosplenial and adjacent motor cortex; G9, cingulate and adjacent motor cortex. W1–W3, white matter scoring regions: W1, cerebellar white matter; W2, mesencephalic tegmentum; W3, pyramidal tract. vCJD, variant Creutzfeldt-Jakob disease.

We generated vacuolation profiles for each mouse line/inocula combination. In RIII and C57BL/6J mice, we observed moderate to mild vacuolation in the medulla and hypothalamus; C57BL/6J mice also exhibited mild vacuolation in the cerebellar peduncle (Figure 2, panels A, B). VM mice showed mild to moderate vacuolation in the medulla, thalamus, and septum, but typically not in the hypothalamus (Figure 2, panel C). Although the distribution of vacuolation was similar between isolates in the different mouse lines, the intensity of vacuolation distribution varied. This difference was most evident in the VM mice, in which the transmission from patient 2 appeared to have a lower intensity of vacuolation than that of patient 1 and the UK patient.

We observed a widespread accumulation of abnormal prion protein (PrP) throughout the brains of inoculated mice, with greatest accumulations apparent in the medulla, hypothalamus, and thalamic areas. We observed variability in the intensity of PrP accumulation between mice both within and between groups. Fine punctate/punctate deposits were the most common form of PrP accumulation in the mice; however, subtle differences were apparent in the hippocampus. In RIII and C57BL/6J, we observed a characteristic PrP deposition in the CA2 region of the hippocampus, whereas VM exhibited PrP deposition in the molecular dentate gyrus with occasional small plaques present in the corpus callosum (Technical Appendix Figure 1).

Biochemical analysis of inocula confirmed the presence of protease-resistant prion protein (PrP^res^) in each of the 3 isolates. We identified a similar PrP^res^ type in isolates from the Spain patients that resembled that of the UK patient and the vCJD diagnostic standard, with a banding pattern dominated by the diglycosylated fragment of the protein and an unglycosylated fragment of ≈19 kDa (Technical Appendix Figure 2). PrP^res^ was readily detected in the brain of RIII and VM mice challenged with all 3 isolates. We identified a similar glycosylation pattern in both mouse lines; a dominant diglycosylated fragment of ≈30 kDa and an unglycosylated fragment of ≈20k Da. We found no apparent differences between the different mouse line/inocula combinations (Technical Appendix Figure 3).

## Conclusions

This transmission study of central nervous system tissue from 2 first-degree relatives with vCJD confirms that the same infectious transmissible spongiform encephalopathy (TSE) agent was responsible for both cases. Comparisons of incubation period, TSE neuropathology, and PrP^res^ biochemistry indicate that this strain is consistent with that of a UK case of vCJD and with historical vCJD transmission data (*6*). The epidemiologic investigation of the 2 related patients indicated that they had shared a common residence and dietary habits, including cattle brain consumption, for >30 years. This finding suggests a common source of infection linked to the consumption of high-risk material in a known BSE area, and these transmission studies support the hypothesis that consumption of BSE-contaminated food products is a major risk factor for vCJD (*7*).

A feature of the UK vCJD epidemic was the relatively young age of the patients at onset. During 1995–2014, only 6 of 177 cases of vCJD identified in the United Kingdom were in persons >55 years of age at the onset of symptoms. Clinical phenotypes in these 6 patients were less consistent than those observed in younger patients (*8*). The evidence suggests that age is not a barrier to either infection or developing the disease; diagnosis of vCJD may become more important as exposed populations become older. Our study demonstrates that older persons harbor the vCJD agent in the central nervous system in a similar manner to younger persons. Small differences in incubation periods and the intensity of TSE vacuolation are apparent, which may be indicative of variation in the titer of the isolates. It is unknown when the 2 patients from Spain were infected, but if they were exposed at the same time, the 23-year difference in age at time of exposure may have influenced pathogenesis and the ability of the agent to replicate. A delay in neuroinvasion or slower rates of replication in the brain could explain why clinical symptoms are more variable in older patients. 

Although our study demonstrates that clinical presentation and infective titer may differ between patients, the overall strain characteristics remain similar. Thus, the vCJD strain can be identified using our strain typing panel regardless of these variable factors.

This study highlights the need for awareness of vCJD in older age groups, particularly in patients with clinical manifestations of atypical dementias. These findings add additional supporting evidence to the hypothesis that a single strain of TSE agent is responsible for vCJD cases, regardless of geographic origin or age at infection, and indirectly support the hypothesis of a dietary origin for primary cases of vCJD.

Technical Appendix Immunohistochemical and Western blot analyses for study of transmission of Creutzfeldt-Jakob disease in wild-type mice from inoculation of brain tissue homogenates from 2 patients from Spain and a reference patient from the United Kingdom.
