# Supplementary material for: Similarities of Variant Creutzfeldt-Jakob Disease Strain in Mother and Son in Spain to UK Reference Case
Source: Emerg Infect Dis. 2017 Sep;23(9):1593–6. doi: 10.3201/eid2309.170159 (PMC5572887; doi:10.3201/eid2309.170159)
Supplement: Technical Appendix — Immunohistochemical and Western blot analyses for study of transmission of Creutzfeldt-Jakob disease in wild-type mice from inoculation of brain tissue homogenates from 2 patients from Spain and a reference patient from the United Kingdom. [file 17-0159-Techapp-s1.pdf]

Article DOI: https://doi.org/10.3201/eid2309.170159

Similarities of Variant Creutzfeldt-Jakob Disease Strain in Mother and Son in Spain to UK Reference Case

Technical Appendix

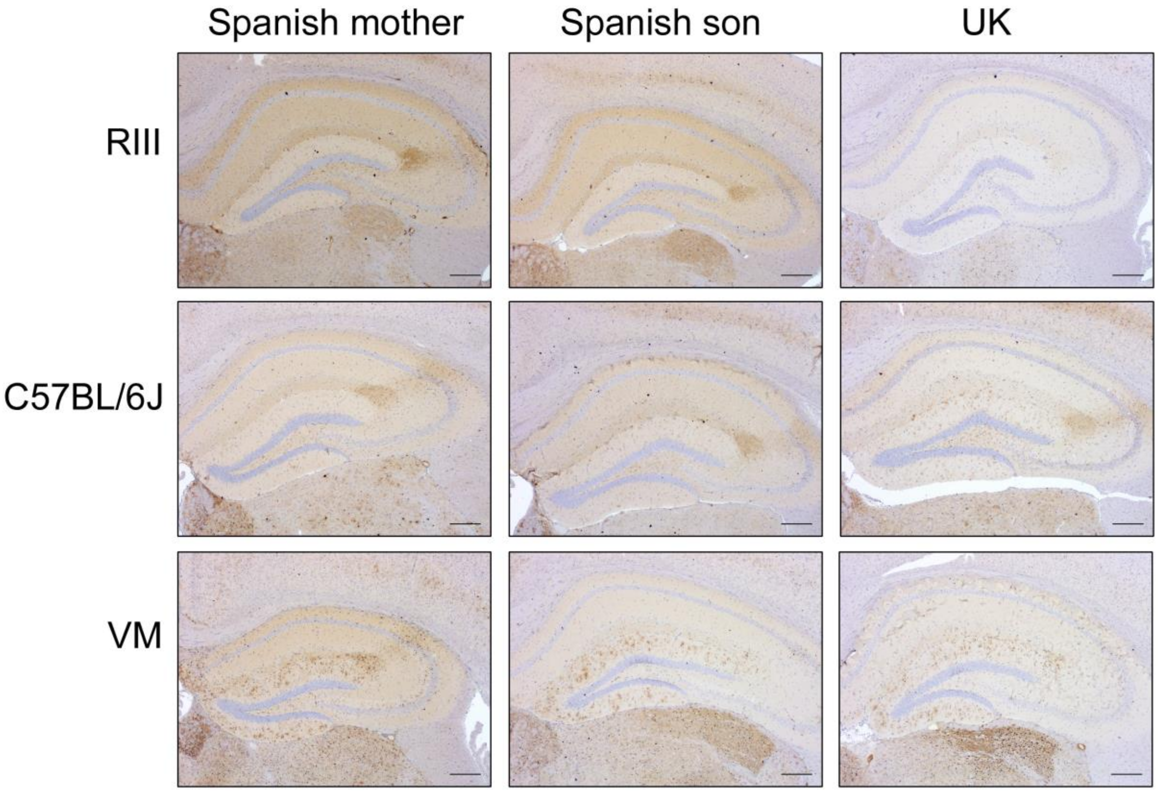

Technical Appendix Figure 1. Immunohistochemical detection of abnormal prion protein in the hippocampus of RIII, C57BL/6J, and VM mice after inoculation with variant CJD brain material from 3 sources. The anti–prion protein detection antibody used was 6H4. Scale bars = 200µm.

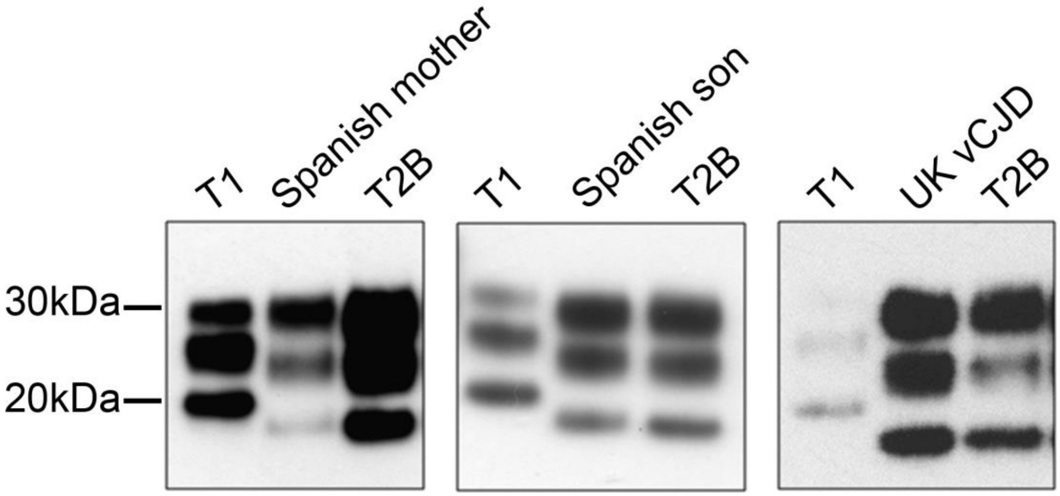

Technical Appendix Figure 2. Western blot analyses of protease resistant isoforms of PrP (PrP^{res}) show similarities between 3 patients with vCJD. Extracts of brain homogenate prepared from frozen frontal cortex tissue of post-mortem samples from 2 Spanish patients and a UK reference case. In each blot, samples are flanked between a sCJD MM1 diagnostic reference sample (T1) and vCJD diagnostic reference sample (T2). Blots were probed with monoclonal antibody 3F4. Blots were probed with monoclonal antibody 6H4. Approximate molecular mass is shown in kDa.

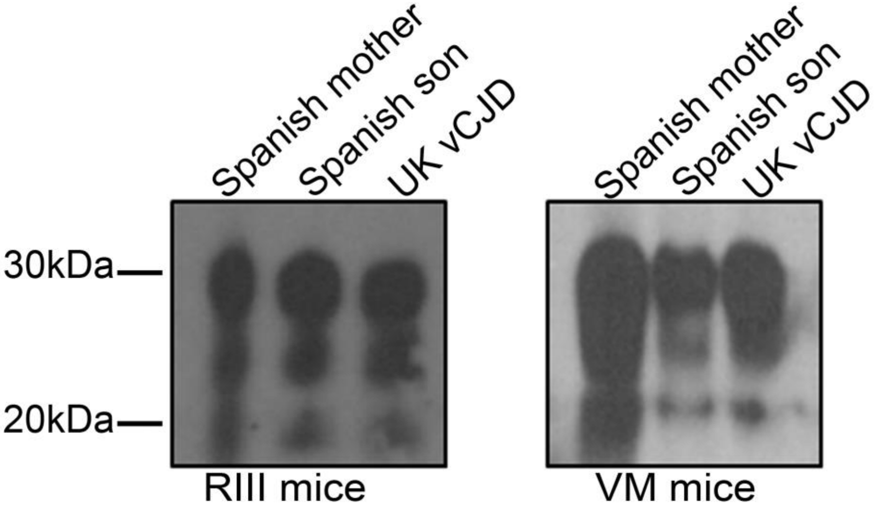

Technical Appendix Figure 3. Western blot analyses of protease resistant isoforms of PrP (PrP^{res}) show similarities between wild type mice inoculated with vCJD infected brain tissue. PrP^{res} in extracts of brain material from RIII and VM wild-type mice inoculated with vCJD brain isolates from 2 Spanish patients and a UK reference case. Blots were probed with monoclonal antibody 6H4. Approximate molecular mass is shown in kDa.
